# Quantitative evaluation of malignant gliomas damage induced by photoactivation of IR700 dye

**DOI:** 10.1080/14686996.2016.1205936

**Published:** 2016-08-22

**Authors:** Morito Sakuma, Sayaka Kita, Hideo Higuchi

**Affiliations:** ^a^ Department of Physics, Graduate School of Science, The University of Tokyo, Tokyo, Japan

**Keywords:** Gliomas, therapy resistance, cell death, photoreactive dye, 60 New topics/Others, 211 Scaffold/Tissue engineering/Drug delivery

## Abstract

The processes involved in malignant gliomas damage were quantitatively evaluated by microscopy. The near-infrared fluorescent dye IR700 that is conjugated to an anti-CD133 antibody (IR700-CD133) specifically targets malignant gliomas (U87MG) and stem cells (BT142) and is endocytosed into the cells. The gliomas are then photodamaged by the release of reactive oxygen species (ROS) and the heat induced by illumination of IR700 by a red laser, and the motility of the vesicles within these cells is altered as a result of cellular damage. To investigate these changes in motility, we developed a new method that measures fluctuations in the intensity of phase-contrast images obtained from small areas within cells. The intensity fluctuation in U87MG cells gradually decreased as cell damage progressed, whereas the fluctuation in BT142 cells increased. The endocytosed IR700 dye was co-localized in acidic organelles such as endosomes and lysosomes. The pH in U87MG cells, as monitored by a pH indicator, was decreased and then gradually increased by the illumination of IR700, while the pH in BT142 cells increased monotonically. In these experiments, the processes of cell damage were quantitatively evaluated according to the motility of vesicles and changes in pH.

## Graphical abstract

**Figure:**
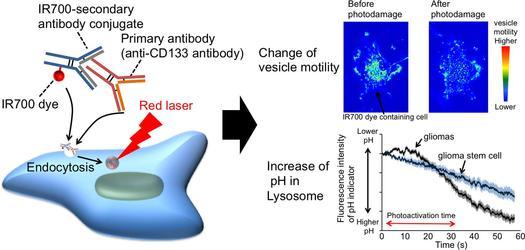

Phototoxicity of IR700 dye conjugated with anti-CD133 antibody to gliomas was evaluated quantitatively by the measurement of vesicle fluctuation and pH indicator.

## 1. Introduction

Malignant gliomas show high invasiveness and spread rapidly via metastasis. Radiotherapy and chemotherapy have been used to eliminate tumor cells because surgical removal of gliomas is often difficult.[1–4] However, tumor cells that survive therapy have a high probability of forming secondary tumors. In particular, recent studies showed that gliomas contain cancer stem cells that are resistant to therapy.[5–7] Therefore, these stem cells are assumed to be one of the main causes of gliomas recurrence,[8,9] and selective removal of cancer stem cells is crucial for gliomas therapy.

Drug delivery systems (DDS) and photodynamic therapy (PDT) have greatly advanced the selective removal of tumor cells with high specificity and low adverse effects.[10–14] 5-aminolevulinic acid (5-ALA) was used to image the area of malignant gliomas during a surgery.[15] Malignant gliomas have been treated with various photosensitizers such as Photofrin®, Laserphyrin®, and Foscan®. These phototoxic dyes were specifically delivered to malignant tumors via an enhanced permeation and retention effect. To enhance the specificity of these treatments for cancer cells, medicines and dyes are often labeled with a cancer antibody. One such phototoxic dye, IR700, is activated by infrared light that transmits well in tissues.[16,17] Recently, clinical trials investigating the photoimmunotherapy effect of IR700 labeled with the antibody have been initiated in the U.S.A. To maximize the specificity and removal of cancer cells by IR700, it is very important to quantitatively evaluate the processes of cell damage and cell death. However, the details of these processes have not been clearly defined.

In this study, we quantitatively evaluated the photoimmunotherapy effects of IR700 on tumor cell damage. IR700 was conjugated to an anti-CD133 antibody, which specifically targets malignant gliomas and stem cells are endocytosed into the cells. The process of cell damage was evaluated by image analysis obtained by phase-contrast and fluorescence microscopy. This study developed a new and easy method for the quantitative evaluation of cell activity and damage through the measurement of intensity fluctuation in phase-contrast images. Specifically, acidic vesicles containing IR700 were damaged by the activation of IR700, causing permeabilization of the vesicle membrane, which resulted in cell death.

## 2. Experimental section

### 2.1. Materials

Near-infrared fluorescent dye, IRDye 700DX NHS ester (IR700), was purchased from Li-COR Bioscience. LysoTracker Yellow HCK-123, pHrodo Green AM (Intracellular pH Indicator), and anti-rabbit IgG (H+L) labeled with AlexaFluor 568 were purchased from ThermoFisher Science. PKH26 Linker Kit was purchased from Sigma-Aldrich Co. LLC. Rabbit anti-CD133 antibody and goat anti-Rabbit IgG (H+L) were purchased from Biorbyte and ThermoFisher Science, respectively. The Annexin-V-Cy5 Apoptosis Detection Kit was purchased from BioVision, Inc. Ethidium homodimer-1 (EthD-1), paraformaldehyde, sodium dihydrogen phosphate dihydrate and sodium dihydrogen phosphate were purchased from Wako Pure Chemicals. Glioma stem cells (BT142 class 3) and gliomas (U87MG, class 4) were purchased from the American Type Culture Collection (ATCC). NeuroCult NS-A basal medium and proliferation supplement were purchased from VERITAS, and platelet-derived growth factor (PDGF), epidermal growth factor (EGF) and fibroblast growth factor (FGF) were from PeproTech Inc. Dulbecco’s modified Eagle’s medium (DMEM) was purchased from Sigma, and Dulbecco’s modified phosphate-buffered saline (PBS) was purchased from Takara Bio Inc. Fetal bovine serum (FBS) was purchased from GIBCO, penicillin-streptomycin was purchased from Wako Pure Chemicals, and the TrypLE express enzyme was purchased from ThermoFisher Science. Glass-bottom dishes were purchased from Matsunami Glass Ind., Ltd.

### 2.2. Conjugation of IR700 with anti-CD133 antibody

IR700 and the anti-CD133 antibody were conjugated using the protocol described by Mitsunaga et al. [16]. IR700 (11.3 μg or 5.8 nmol resolved in DMSO to a concentration of 2.5 mol/L) and goat anti-rabbit IgG (121.8 μg or 0.8 nmol resolved in PBS to a concentration of 13.3 μmol/L) were reacted in a solution containing 10 mM Na_2_HPO_4_ (pH: 8.0) for 2 h at room temperature. Then, the IR700 and anti-rabbit IgG complex was mixed with the rabbit anti-CD133 antibody to prepare the conjugate of IR700, anti-rabbit IgG and anti-CD133 (IR700-CD133).

IR700-CD133 specifically bound to gliomas (BT142 and U87MG) expressing CD133 on the cell membrane,[18] while the conjugate did not bind to neuron-like Cath.a-differentiated cells (Supplemental material, Figure 1(a) and (c)). IR700-CD133 also did not bind to breast cancer cells, MDA-MB-231 and KPL-4, that over-expresses HER1 (MDA), HER2 (MDA and KPL) and PAR1 (MDA) (Supplemental material, Figure 1(d)-(f)). The U87MG are labeled with IR700-CD133 but not with uncoupled-IR700 (Supplemental material, Figure 1(a) and (b)). These results indicate that CD133-IR700 conjugate bound specifically to the CD133-expressing cells.

**Figure 1.**
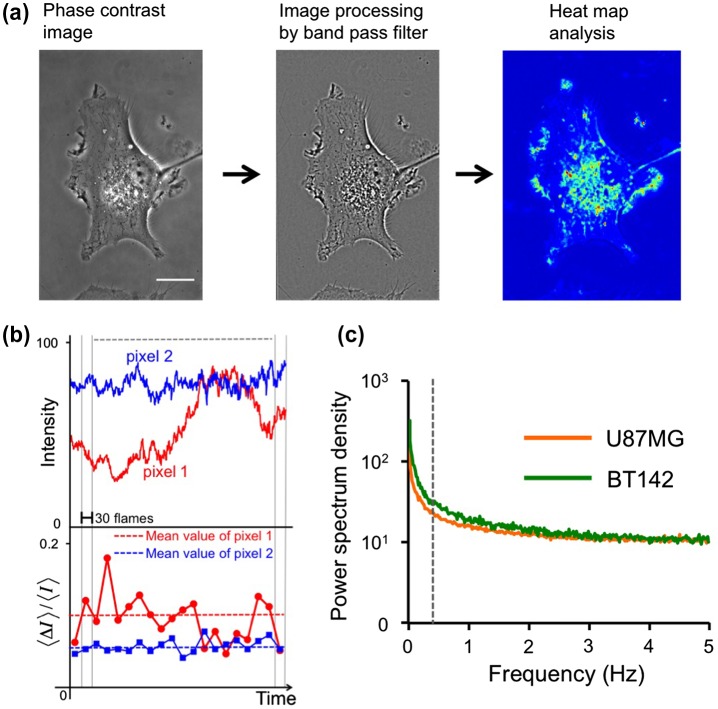
The calculation of intensity fluctuation in U87MG and BT142 cells. In the left panel in (a), phase contrast images are shown. In the middle panel in (a), the image was processed by a band pass filter from 390 to 1,300 nm. In the right panel in (a), the intensity fluctuation of binned pixels is shown as a heat map. Scale bar: 10 μm; (b), calculation of the intensity fluctuation. The upper graph represents the intensity change in two pixels. Lower graph, the fluctuations,<ΔI>/<I>, in binned pixels were averaged over 30 frames. Dashed lines show the mean values for 20 data points in 1 m; (c), power spectrum densities were calculated in the region shown in the images. The y-axis is a scale and relative to the density at 5 Hz. Vertical bar indicates the frequency at 0.33 Hz.

### 2.3. Preparation of gliomas

BT142 cells were cultured and formed neurospheres in medium-BT (NS-A basal medium supplemented with 10% V/V proliferation supplement, 20 ng/mL EGF, 20 ng/mL FGF, 100 ng/mL PDGF, 2 μg/mL heparan sulfate and 100 U/mL penicillin). The cells were triturated with a micropipette to produce a single-cell suspension. The single cells were seeded onto glass-bottom dishes at a concentration of 1.0 × 10^4^ cells/mL. Adherent U87MG cells were cultured in medium-U (DMEM supplemented with 10% FBS, 100 U/mL penicillin). After establishing a confluent culture at 37 °C with 5% CO_2_ and 95% air, the cells were recovered by treatment with the TrypLE express enzyme (1 ×). Recovered cells were seeded at a concentration of 1.0 × 10^4^ cells on a glass-bottom dish coated with 2% collagen, and cells were incubated for one day in medium-BT after initial adhesion.

### 2.4. Preparation of gliomas

U87MG and BT142 cells from the glass-bottom dishes were loaded with 10 and 40 nM of IR700-CD133 in medium-BT and incubated for 1 h at 37 °C with 5% CO_2_ and 95% air. After washing with medium, the dishes were placed on a microscope stage with a controlled temperature and CO_2_ concentration.

### 2.5. Fluorescence microscopy of gliomas

Fluorescence images of gliomas in experiments with IR700, LysoTracker and a pH indicator were obtained using an inverted microscope (IX-70, Olympus) equipped with a phase-contrast objective (Olympus, PLAPON 60XOPH), a spinning disk confocal unit (CSU-10, Yokogawa), EMCCD cameras and an incubator (TOKAI HIT Co., Ltd.) to control the temperature at 37 °C and the CO_2_ concentration at 5% in the glass-bottom dishes. For cell damage experiments, the cells were illuminated with a red laser (635 nm, 100 mW, EDMUND OPTICS) for 10 s and 30 s to photoactivate IR700. The fluorescence images of IR700 excited by the red laser were captured with an EMCDD camera (iXon 3, Andor Technology Ltd.) equipped with a 690–730 bandpass filter. After laser treatment, the cells were incubated with 5.0 nM EthD-1 and 1 μL Annexin-V-Cy5 to detect cell death. Epi-fluorescence images of EthD-1 and Annexin-V excited by the blue (20 mW, Showa Optronics) and green lasers (50 mW, Showa Optronics), respectively, were captured with EMCCD cameras.

Confocal images of LysoTracker and IR700 excited by the blue and red lasers were captured simultaneously by two EM-CCD cameras (iXon 3 and iXon+, Andor Technology Ltd.) at a rate of 10 frames/s. The confocal images of the pH indicator excited by the blue laser were observed with the EMCCD camera during irradiation of IR700 by the red laser.

### 2.6. Calculation of intensity fluctuation to evaluate cell damage

The motility of the vesicles in the cells changed as the cell damage progressed. The motility of the vesicles was measured by the fluctuation of intensity in phase-contrast images of cells obtained by illumination with a halogen lamp. The phase-contrast images of cells were obtained for 60 s at a rate of 10 frames/s. The images were processed with a special band-pass filter from 390 nm to 1,300 nm to enhance the structure of the vesicles with ImageJ software (center panel in Figure 1(a)). Then, the number of pixels in the filtered images was reduced by binning 4 × 4 pixels. The intensity fluctuation of binned pixels for a given time was calculated by the standard deviation (<ΔI>) of the intensity divided by the mean intensity (<I>) (upper panel in Figure 1(b)). The power spectra of the intensity decreased steeply at <0.33 Hz (Figure 1(c)). Spectra <0.33 Hz indicated cell motility. To avoid cell motility, the fluctuations,<ΔI>/<I>, in binned pixels were averaged over 30 frames or 3 s. Then, 20 fluctuation data points were averaged over 1 m to reduce the noise (lower panel in Figure 1(b)). Finally, fluctuation values averaged over 1 m in all binned pixels are shown by a heat map (right panel in Figure 1(a)).

## 3. Results

### 3.1. Tumor cell damage and death induced by IR700 phototoxicity

**Figure 2.**
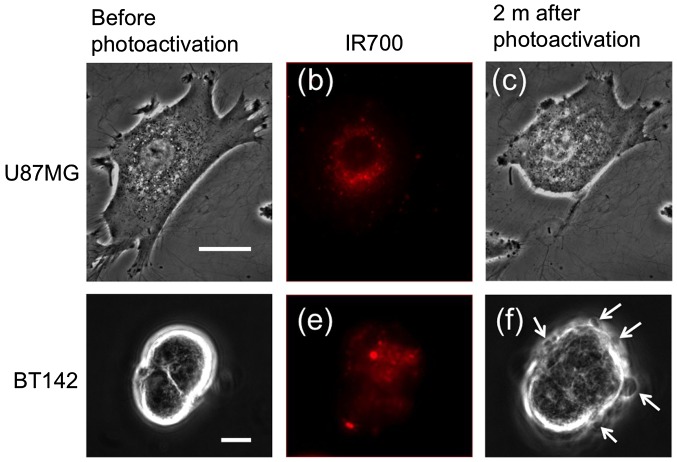
The change in cell shape by photoactivation of IR700; (a), (c), (d) and (f) show phase-contrast images of U87MG and BT142 cells before and after photoactivation for 30 s. (b) and (e) show the fluorescence of IR700 in the cells corresponding to (a) and (d). White arrows in (f) show bleb structures on the membrane of BT142 cells. Scale bar: 10 μm.

We investigated the changes in cell shape induced by phototoxicity of IR 700 activated by the red laser. IR700 was first reacted with goat anti-rabbit IgG as the secondary antibody, as used in immunocytochemistry. Then, the IR700-anti-rabbit complex was bound to the rabbit anti-CD133 antibody as the primary antibody (IR700-CD133). Using this method, the primary antibody could bind the target cell without changing the IR700-anti-rabbit complex. U87MG and BT142 cells were loaded with 40 nM of IR700-CD133 and incubated for 1 h at 37 °C. Adhesive U87MG cells bound to the glass and extended lamellipodia and filopodia (Figure 2(a)). In contrast, BT142 cells showed no focal adhesion to the glass substrate and formed neurospheres (Figure 2(d)). IR700 fluorescence was observed under a fluorescence microscope (Figure 2(b) and (e)). IR700 was predominantly distributed in vesicles near the nucleus in U87MG cells. After photoactivation by the red laser for 30 s, extensions of U87MG cells retracted and the cell body gradually shrank (Figure 2(c)). Photoactivation induces cellular damage through reactive oxygen species (ROS) and the heat generated by IR700.[16,17] Spherical BT142 cells swelled and formed blebs after photoactivation; when these blebs were produced in one region of the cell membrane, those in the other regions shrank (white arrows in Figure 2(f)). The changes in cell shape, bleb formation and swelling continued for > 30 m. These results indicate that IR700-CD133 conjugates are internalized by and damage the cells through the photoactivation of IR700.

The cell was stained with Annexin-V and EthD-1 to classify the modes of cell death such as apoptosis or necrosis. Annexin-V specifically adsorbs to phosphatidylserine on the cell membrane of apoptotic cells. In live cells, phosphatidylserine on the outer layer of the cell membrane is flip-flopped to the inner layer of the cell membrane by phospholipid translocase, resulting in most of the phosphatidylserine localized to the inner layer.[19] However, in the early stages of apoptosis and later necrosis, phosphatidylserine is exposed on the outer layer of the membrane, presumably through the inactivation of phospholipid translocase and activation of scramblase. Figure 2(a) and (b) show the fluorescence images of damaged cells stained with Annexin-V-Cy5. The cells were loaded with 40 nM IR700-CD133 conjugates and photoactivated for 30 s. U87MG cells showed limited Annexin-V staining at 60 m after photoactivation. In contrast, the membrane of BT142 cells was stained by Annexin-V. The ratio of cells stained by Annexin-V to total cells with 40 nM of IR700-CD133 and photoactivation for 30 s is shown in Supplemental material, Table 1. The ratio increased from 10% to 30% in U87MG and from 61% to 85% in BT142 at 30 m and 60 m, respectively, after the photoactivation. These results suggest that BT142 and U87MG are Annexin-V-positive and negative, respectively, within 60 m.

**Figure 3.**
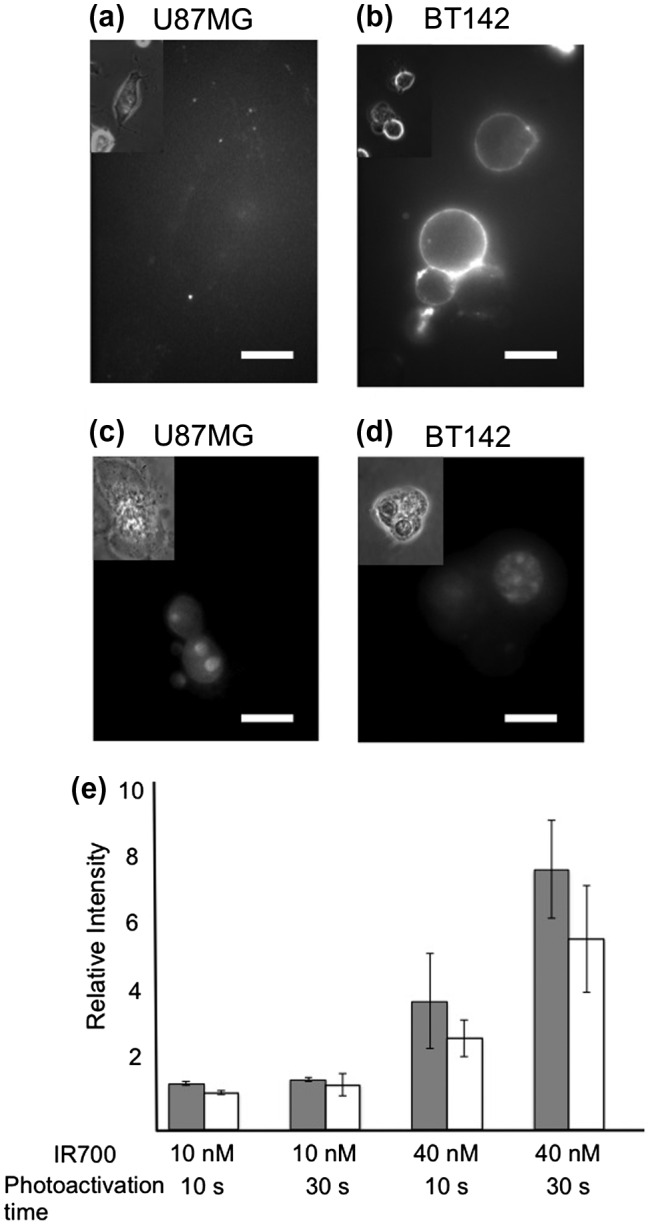
The evaluation of damage and cell death in U87MG and BT142 cells; (a) and (b), the nucleus in the cells stained by EthD-1 at 20 m after photoactivation of IR700. Inset images are phase-contrast images of the corresponding cells; (c) shows the fluorescence intensity of EthD-1 after photoactivation relative to that before photoactivation. The numbers represent the data from six cells; (d) and (e) show fluorescence images of Annexin-V 20 m after photoactivation with loading 40 nM of IR700-CD133 in cells. Scale bar: 10 μm.

To evaluate the mode of necrotic cell death, the nucleuses of damaged cells were stained by ethidium homodimer-1 (EthD-1). EthD-1 permeates into the cell and the nucleus when the permeability of the cell membrane is increased by cell damage. The fluorescence intensity of the cytoplasm gradually increased, and staining in the nucleus was observed at 20 m after photoactivation (Figure 3(c) and (d)). The relative fluorescence intensity of EthD-1 permeabilized in U87MG and BT142 cells was measured in the nucleus, and this intensity depended on the amount (10 nM and 40 nM) of IR700-CD133 conjugate and the photoactivation time (10 s and 30 s) (Figure 3(e)). Loading of 10 nM IR700 resulted in a limited increase in fluorescence intensity after photoactivation, indicating that 10 nM IR700 was not sufficient to enhance cell permeability. The ratio of cells stained by EthD-1 to total cells with 40 nM of IR700-CD133 and photoactivation for 30 s was also measured (Supplemental material, Table 1). The ratio increased from 78% to 89% in U87MG and from 22% to 56% in BT142 at 10 m and 20 m, respectively, after the photoactivation, indicating that both cells are EthD-1 positive.

We observed the morphology of a nucleus stained by EthD-1 and cell shape at 2 h after photoactivation of IR700 to confirm the modes of cell death. The membranes of both cells were ruptured, and the effluent of vesicles was observed in the region swelled by the cell damage (Supplemental material, Figure 2(b) and (e)). The fragmentation of the nucleus was not observed (Supplemental material, Figure 2(c) and (f)). These results indicate that both cells progressed in necrotic cell death.

### 3.2. Analysis of the fluctuation of vesicle movements in the process of cell damage

**Figure 4.**
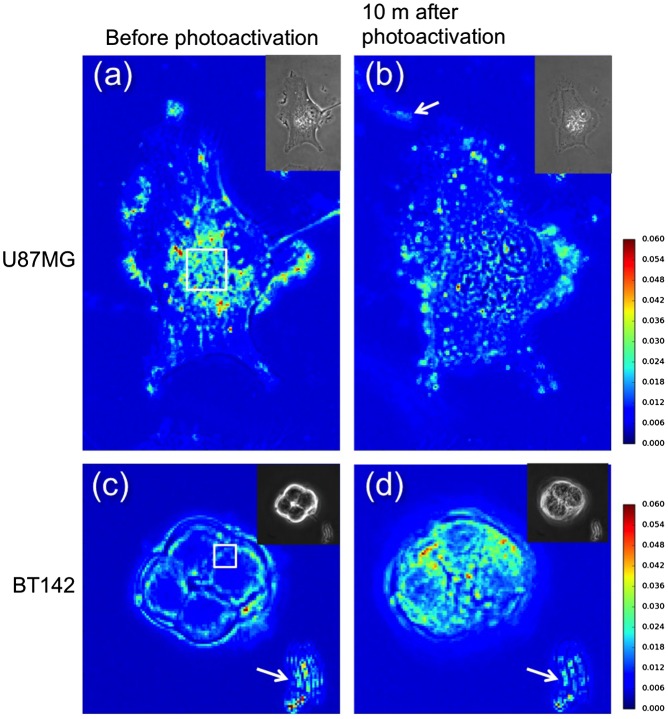
The mean values of intensity fluctuation in cells for one minute shown by a heat map; (a) and (c) show results obtained before photoactivation; (b) and (d) show the results after photoactivation. The cells were loaded with 40 nM of IR700-CD133 and photoactivated for 30 s. The bright line marked by a white arrow indicates the fluctuation of dust but not a cell. White rectangles indicate the area used for calculating the fluctuation in Figure 5.

The motility of vesicles was affected by photoactivation of IR700. The motility gradually decreased in U87MG cells, but not in BT142 cells, following photoactivation of IR700. The density of vesicles near the nucleus was too high to detect the position of individual vesicles. However, the change in intensity in image pixels was detectable even at a high density of vesicles. Therefore, the fluctuation of the intensity in pixels on cell images was calculated using newly developed software; these fluctuation values are presented as heat maps (Figure 4). A highly fluctuating area in U87MG cells was distributed close to the nucleus prior to photoactivation (Figure 4(a)). The area of fluctuation was also distributed near the periphery of the cells, due to the active movements of lamellipodia and filopodia. The centroid of cells barely changed throughout the observation period; thus, the area of fluctuation near the cell nucleus predominantly displayed vesicle movement without the movement of the whole cell (Supplemental material, Movies 1–4). The area of fluctuation in U87MG cells greatly decreased with the decrease in vesicle movement 10 m after photoactivation (Figure 4(b)). In addition, ruffling of the pseudopodium almost stopped, and thinner pseudopodia often shrank. The area of high fluctuation in BT142 cells was limited to the area around the nucleus and was much smaller than that in U87MG cells (Figure 4(c)); the majority of the cellular space was occupied by the nucleus, with less space for the vesicles to move. The area of fluctuation in BT142 cells gradually increased after photoactivation.

**Figure 5.**
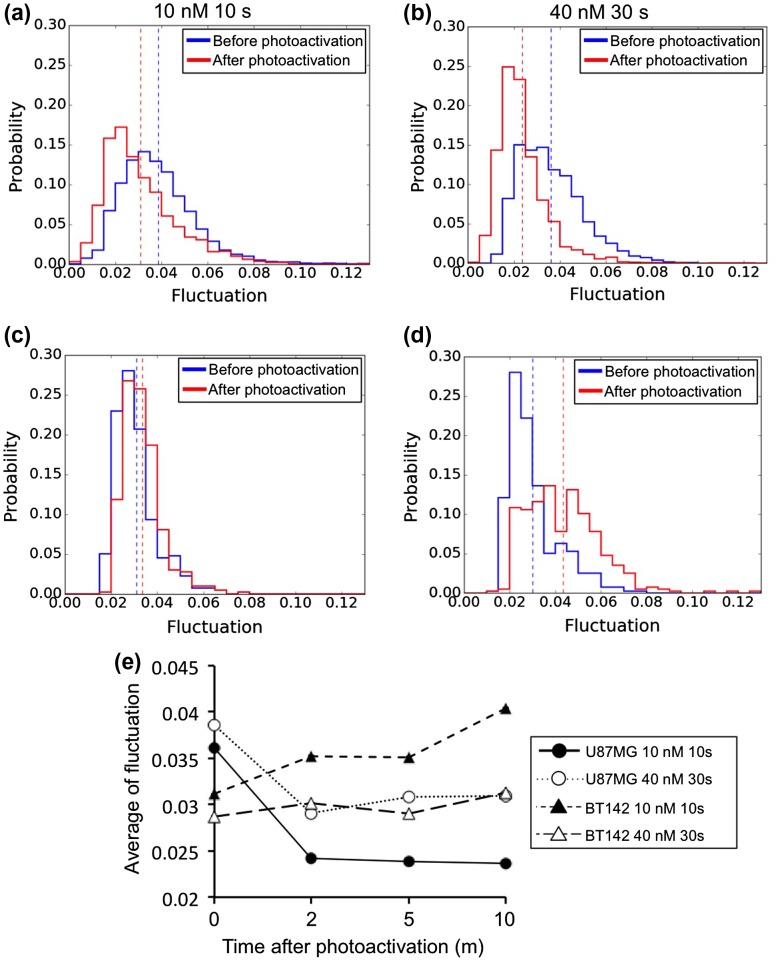
The distribution of intensity fluctuation. The fluctuation was calculated in the regions of interest shown in rectangles in Figures 4(a) and (c). Parts (a) and (b) show the intensity fluctuation in U87MG cells loaded with 10 nM and 40 nM of IR700-CD133 and photoactivation times of 10 s and 30 s; (c) and (d) show the fluctuation in BT142 cells. Dashed lines show the mean values of histograms. The histograms of U87MG and BT142 cells display values obtained for six cells; (e) shows the time course of the mean values of the histograms.

The distribution of intensity fluctuation (ΔI/I) was quantitatively evaluated in the region of interest. The regions (13 × 13 μm and 6.24 × 6.24 μm for U87MG and BT142 cells, respectively) near the nucleus were selected for the calculation, as most vesicles were distributed there (Figure 4(a) and (c)). The total numbers of binned pixels, 0.52 × 0.52 μm in size, contained in the regions were 625 (= 25 × 25) and 144 (= 12 × 12) for U87MG and BT142 cells, respectively. The intensity fluctuations of the binned pixels were calculated by the method shown in Figure 1, 10 m after photoactivation for 10 s and 30 s at IR700 concentrations of 10 nM and 40 nM (Figure 5). The histogram of the fluctuation showed that the probability increased and then decreased with the magnitude of fluctuation (Figure 5). The mean values of the histogram in U87MG cells decreased from 0.039 to 0.031 at 10 nM IR700 (Figure 5(a)) and from 0.036 to 0.023 at 40 nM (Figure 5(b)) following photodamage. In contrast to this change, the mean values in BT142 cells increased from 0.031 to 0.034 at 10 nM (Figure 5(c)) and from 0.031 to 0.040 at 40 nM (Figure 5(d)) following photodamage. The standard errors of the mean values were smaller than 10^−5^ so that the change in the fluctuation were significant. Figure 5(e) shows the time course of the mean values of the fluctuation after photoactivation. The values in U87MG cells decreased and became almost constant at > 2 m, whereas the fluctuation in BT142 cells gradually increased over time. These results indicate that in U87MG cells, the change in the intensity fluctuation induced by photodamage was the opposite of that in BT142 cells.

### 3.3. Colocalization of acidic organelles and vesicles containing IR700

**Figure 6.**
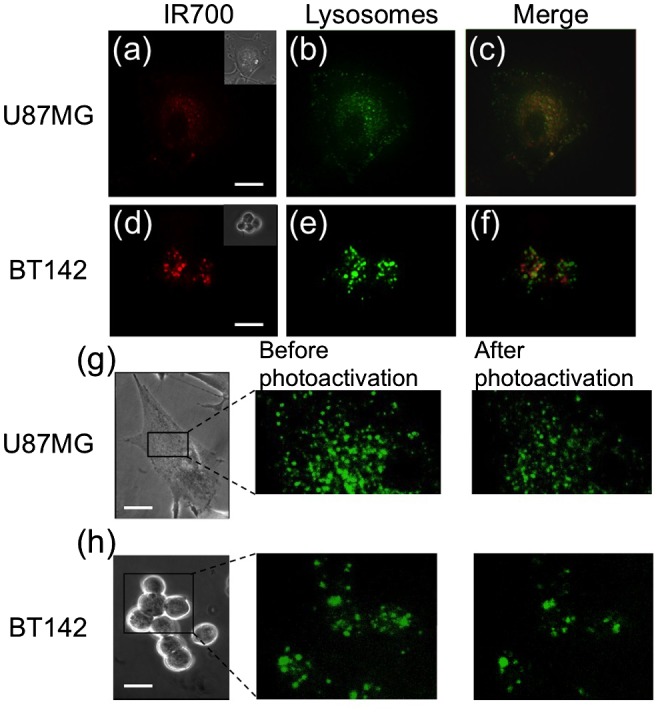
Colocalization of vesicles containing IR700 and lysosomes in U87MG and BT142 cells; (a), (b), (d), and (e) show the fluorescence of IR700 and LysoTracker (50 nM) in U87MG and BT142 cells, respectively. Inset images show phase-contrast images of the cells; (c) and (f) show the merged images of IR700 and LysoTracker; (g) and (h) show the fluorescence of LysoTracker before and after photoactivation for 30 s at 40 nM IR700. Scale bar: 10 μm.

IR700-CD133 was endocytosed into tumor cells and was located near the nucleus (Figure 2(b) and (e)). In general endocytosis, materials outside of the cell are taken into the endosomes, which mature to lysosomes during transport to the nucleus.[20] Thus, endosomes containing IR700-CD133 should mature into lysosomes within approximately 1 h after IR700-CD133 is loaded into the cells. We investigated the localization of lysosomes and vesicles containing IR700 by LyosTracker staining, as the fluorescence intensity increases in acidic lysosomes. IR700 was endocytosed into vesicles and transported to a region near the nucleus (Figure 6(a) and (d)). LysoTracker was detected near the nucleus, as well as in the cell periphery (Figure 6(b) and (e)). IR700 fluorescence (shown by the red color in Figure 6) near the nucleus overlapped with the green fluorescence of LysoTracker (Figure 6(c) and (f)). This finding suggests that most of the vesicles containing IR700 were colocalized with lysosomes.

Figure 6(g) and (h) show the fluorescence images of LysoTracker before and after photoactivation of IR700. The cells were treated with 40 nM of IR700-CD133 for 1 h and then photodamaged for 30 s. The fluorescence intensity of LysoTracker was almost zero in some lysosomes in both cell types 2 m after photoactivation (Figure 6(g) and (h)), while the fluorescence intensity in the lysosomes not treated with IR700-CD133 decreased only slightly by photobleaching (Supplemental material, Figure 3), indicating lysosome damage and leaky lysosomal membranes.

### 3.4. pH changes in damaged cells

**Figure 7.**
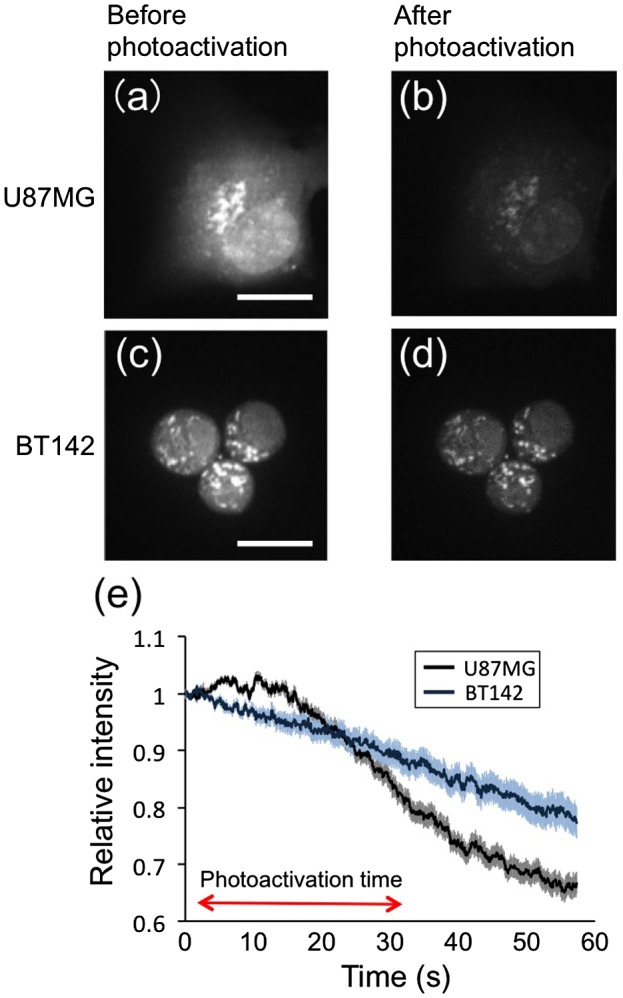
The pH changes in photodamaged U87MG and BT142 cells. Cells were treated with 0.1 μL of a pH indicator, 40 nM of loading IR700-CD133 and a photoactivation time of 30 s; (a)-(d) show the fluorescence changes before and after photoactivation; (e) shows the intensity in photodamaged cells relative to that in cells without loading IR700-CD133. The cells were photodamaged from 2 s to 32 s. The data were obtained from 30 areas of eight U87MG cells and 23 BT142 cells. Scale bar: 10 μm.

To evaluate the process of cell damage, the acidic organelles were stained with a pH indicator, and their localization and intensity of staining were evaluated. The fluorescence intensity of the pH indicator was high in organelles distributed near the nucleus and low in the cytoplasm, indicating a lower pH in late endosomes and lysosomes (Figure 7(a) and (c)). The fluorescence intensity of acidic organelles and the cytoplasm gradually decreased after photoactivation of 40 nM IR700 (Figure 7(b) and (d), Supplemental material, Movies 5–8).

The changes in fluorescence intensity after photoactivation were investigated by calculating the mean intensity in the area of 30 × 30 pixels (U87MG) and 10 × 10 pixels (BT142) near the nuclei for 60 s. The graph shows the fluorescence intensity in photodamaged cells relative to that in cells not loaded with IR700-CD133 (Figure 7(e) and Supplemental material, Figure 4). The relative fluorescence intensity in U87MG cells slightly increased for approximately 10 s and then decreased gradually over time (Figure 7(e)), while the intensity in BT142 cells decreased almost linearly and was higher than that in U87MG cells at >20 s. These results indicate that with the exception of the early stage in U87MG cells, photodamage increased the pH of cells and that this effect was presumably induced by a defect of the lysosomes.

## 4. Discussion

The selective removal of tumor cells has long been anticipated in the medical field. Indeed, IR700 bound to antibodies has been developed for the selective removal of tumor cells by photodamage.[16,17] Here, we determined the type of cell death induced by IR700 phototoxicity and then measured the changes in intensity fluctuation and pH during cell damage to understand the process driving cell death. The intensity fluctuation and pH increased gradually over time after photodamage; however, BT142 cells died by apoptosis, while U87MG cells died by necrosis.

The types of cell death occurring in U87MG and BT142 cells were determined by EthD-1 and Annexin-V-Cy5 staining, and morphological changes. U87MG cells displayed EthD-1 staining, but not by Annexin-V staining, after photoactivation of IR700. The fragmentation of the nucleus detected by EthD-1 was not observed. Moreover, U87MG did not form bleb structures, and vesicles were diffused out of cells at 2 h after photoactivation. These results indicate that the mechanism of death in U87MG cells was necrosis.[21] BT142 cells displayed EthD-1-positive and Annexin-V-positive staining, and formed bleb formation within 60 m after photoactivation. BT142 seemed to have the characteristics of apoptosis and necrosis within 60 m.[21,22] It is consistent with the reported result that photodamage of A431-cells by IR700 induced both apoptosis and necrosis.[23] At 2 h after photoactivation, vesicles in BT142 cells diffused in the same degree as U87MG cells and the fragmentation of the nucleus detected by EthD-1 was not observed, suggesting necrotic cell death. These results indicate that BT142 had characteristics of necrotic and apoptotic cell death in an early stage of cell damage and progressed in necrotic cell death at a later stage.

The vesicle motility inside of cells changed as the cell damage progressed. The majority of the vesicles accumulated at a high density near the cell nucleus, and the movement of individual vesicles near the nucleus could not be tracked because of the high density. We therefore calculated the intensity fluctuation due to the vesicle movement in phase-contrast images using our newly developed software (Figure 1). The vesicle motility or fluctuation in adhesive U87MG cells decreased after photoactivation (Figure 4(a)–(b) and 5(a)–(b)), which can be explained by the observation that motor proteins or anchor proteins bound to vesicles are denatured and proteolyzed by photodamage and by proteases leaking from lysosomes.[24] In contrast, the vesicle fluctuation in spherical BT142 cells gradually increased (Figure 4(c)–(d) and 5(c)–(d)). In BT142 cells, bleb formation and swelling of cells were observed after photoactivation of IR700. Indeed, the cytoskeleton, including microtubules and actin filaments, may be damaged by the influx of culture medium into the cells.[25] These results indicate that different types of cell death lead to different changes in intensity fluctuations (increase in U87MG and decrease in BT142 cells).

To investigate the process of cell death in U87MG and BT142 cells, acidic organelle damage was examined by confocal microscopy. The acidic organelles stained by LysoTracker were colocalized with the vesicles containing IR700 (Figure 6(a)-(f)), and the intensity of LysoTracker in most organelles gradually decreased, indicating that protons in the organelles leaked due to the cellular damage (Figure 6(g) and (h)). The process of acidic organelle damage was evaluated by the real-time observation of pH indicator staining (Figure 7). The fluorescence intensity of the pH indicator in U87MG cells increased and then gradually decreased with photoactivation, while that in BT142 cells decreased (Figure 7(e)). The pH in U87MG cells was presumably decreased by proton leakage from acidic organelles such as lysosomes and late endosomes, and then the pH was increased by protons and proteases leaking from lysosomes. The number of rupturing acidic organelles was not high; thus, photoactivation induced an increase in the permeability of acidic organelle membranes.[24] The decrease in fluorescence of BT142 cells was slower than that of U87MG cells, indicating that BT142 cells have a higher resistance to photodamage than U87MG cells.

Cancer stem cells, such as BT142 cells, show the stem cell-like properties of anaplasticity and multi-differentiation capacity.[5–9,26] The anaplasticity of stem cells is maintained by reducing the level of ROS,[27] as the concentration of ROS-removing enzymes, such as glutathione, is higher in cancer stem cells than normal cancer cells. Thus, BT142 cells likely reduced the ROS levels induced by IR700 photoactivation, and lysosomal damage was reduced compared with U87MG cells. This finding is consistent with the observation that a decrease in fluorescence intensity of the pH indicator in BT142 cells was slower than that in U87MG cells (Figure 7(e)). Thus, a reduction in damage would lead to a higher survival rate of BT142 cells in tumors.

## 5. Conclusion

The processes of glioma (U87MG) and glioma stem cell (BT142) damage were evaluated using a novel method and a pH indicator. A near-infrared fluorescence dye, IR700, was applied to induce cell damage, and the changes in vesicle movement inside the cells induced by photoactivation were measured according to the intensity fluctuation observed in phase-contrast images. The movements or fluctuation in U87MG cells were decreased after photoactivation, while those in BT142 cells were increased. Our new method to evaluate cell damage by intensity fluctuation is easy to use and sensitive enough to detect damage without photobleaching, which occurs with fluorescence dyes. In addition, the pH was changed as a result of damage to acidic organelles such as lysosomes. We found that the pH change in BT142 cells was slower than that in U87MG cells, which indicates that the damage process of BT142 cells is different from that of U87MG cells and that BT142 cells are more resistant to photodamage than U87MG cells. These fluctuation and pH methods are valuable for cell screening in combination with other therapeutic treatments.

## Disclosure statement

No potential conflict of interest was reported by the authors.

## Supplemental material

The supplemental material for this paper is available online at http://dx.doi.org/10.1080/14686996.2016.1205936.

## Supplementary Material

TSTA_1205936_supplemental_files.zip
