# Supplementary figures and images for: Quantitative evaluation of malignant gliomas damage induced by photoactivation of IR700 dye
Source: Sci Technol Adv Mater. 2016 Aug 22;17(1):473–82. doi: 10.1080/14686996.2016.1205936 (PMC5111559; doi:10.1080/14686996.2016.1205936)

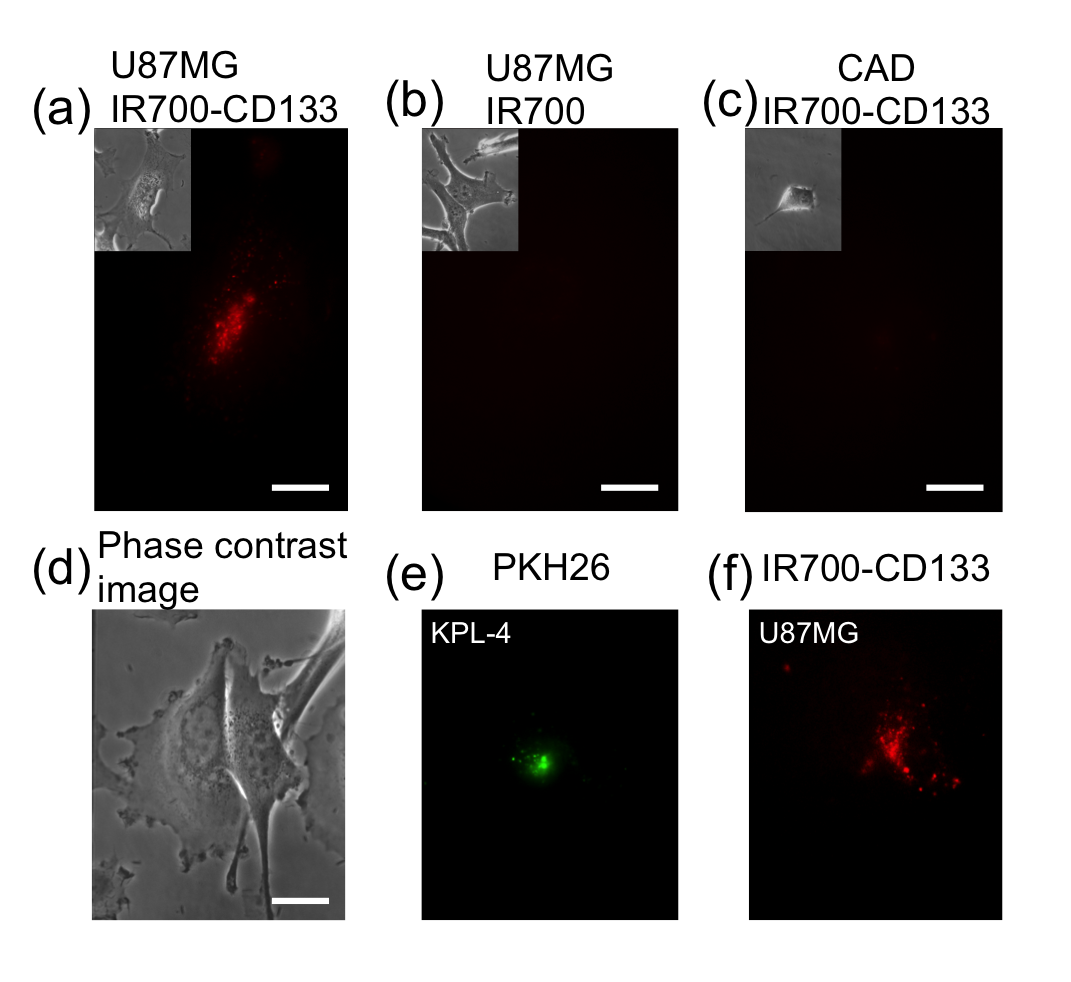

Supplement: TSTA_1205936_supplemental_files.zip [file tsta_a_1205936_sm2050.zip › Supplemental Figure 1.tif]

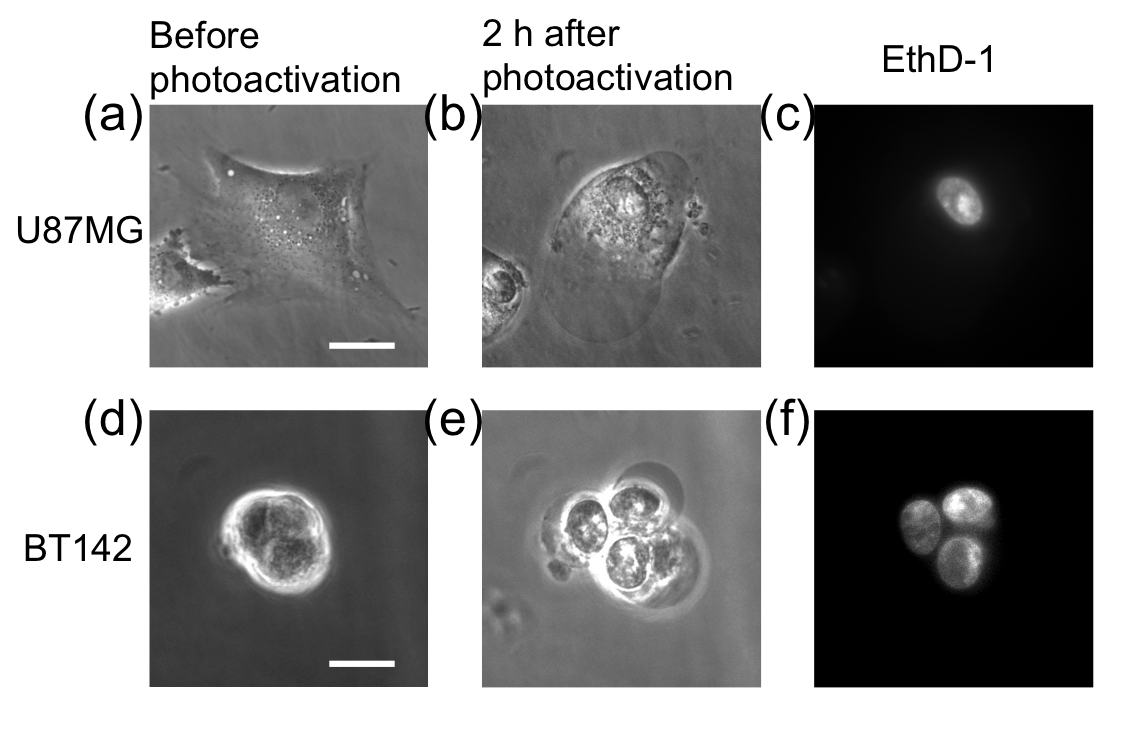

Supplement: TSTA_1205936_supplemental_files.zip [file tsta_a_1205936_sm2050.zip › Supplemental Figure 2.tif]

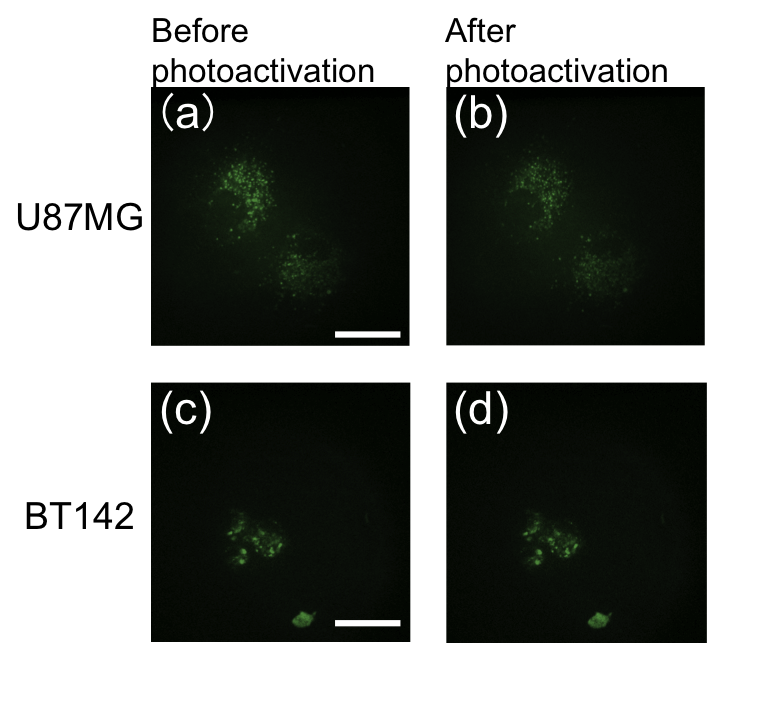

Supplement: TSTA_1205936_supplemental_files.zip [file tsta_a_1205936_sm2050.zip › Supplemental Figure 3.tif]

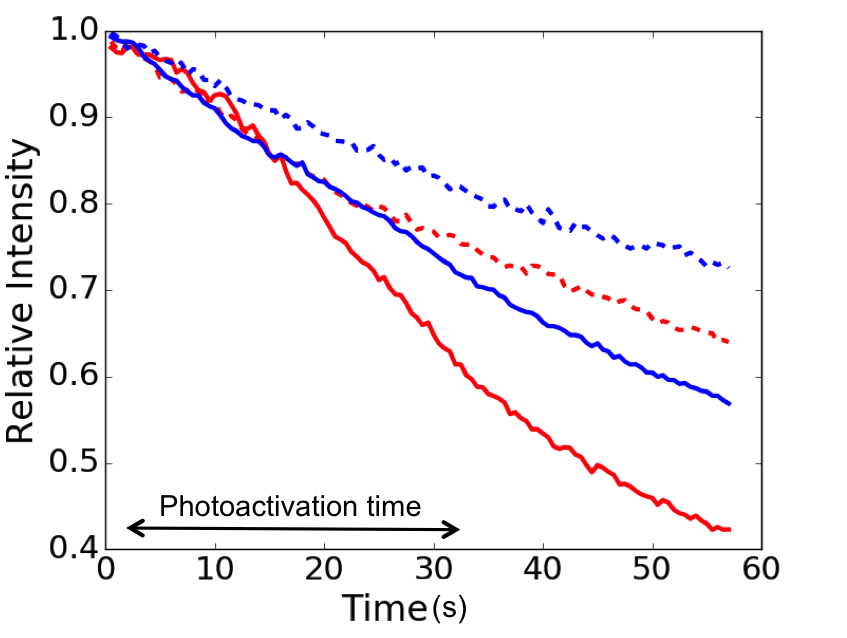

Supplement: TSTA_1205936_supplemental_files.zip [file tsta_a_1205936_sm2050.zip › Supplemental Figure 4.tif]

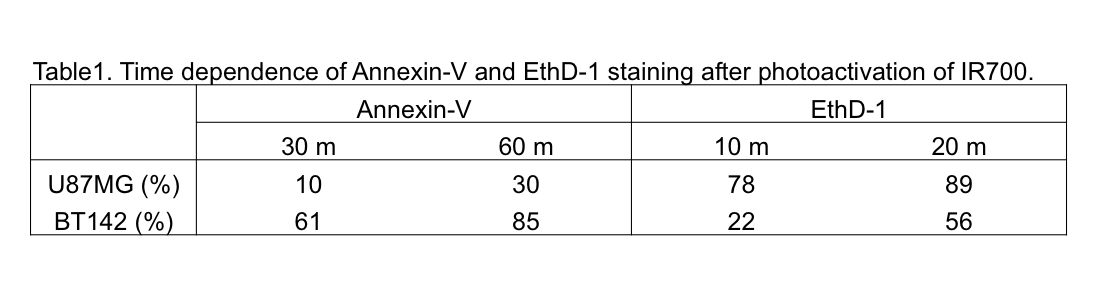

Supplement: TSTA_1205936_supplemental_files.zip [file tsta_a_1205936_sm2050.zip › Supplemental Table1.tif]
